# Salinity Inhibits Rice Seed Germination by Reducing α-Amylase Activity via Decreased Bioactive Gibberellin Content

**DOI:** 10.3389/fpls.2018.00275

**Published:** 2018-03-05

**Authors:** Li Liu, Weili Xia, Haixia Li, Hanlai Zeng, Benhui Wei, Suoyi Han, Changxi Yin

**Affiliations:** ^1^College of Plant Science and Technology, Huazhong Agricultural University, Wuhan, China; ^2^Cash Crops Research Institute, Guangxi Academy of Agricultural Sciences, Nanning, China; ^3^Industrial Crops Research Institute, Henan Academy of Agricultural Sciences, Zhengzhou, China

**Keywords:** α-amylase activity, α-amylase gene expression, gibberellin, rice, salinity, seed germination

## Abstract

Seed germination plays important roles in the establishment of seedlings and their subsequent growth; however, seed germination is inhibited by salinity, and the inhibitory mechanism remains elusive. Our results indicate that NaCl treatment inhibits rice seed germination by decreasing the contents of bioactive gibberellins (GAs), such as GA_1_ and GA_4,_ and that this inhibition can be rescued by exogenous bioactive GA application. To explore the mechanism of bioactive GA deficiency, the effect of NaCl on GA metabolic gene expression was investigated, revealing that expression of both GA biosynthetic genes and GA-inactivated genes was up-regulated by NaCl treatment. These results suggest that NaCl-induced bioactive GA deficiency is caused by up-regulated expression of GA-inactivated genes, and the up-regulated expression of GA biosynthetic genes might be a consequence of negative feedback regulation of the bioactive GA deficiency. Moreover, we provide evidence that NaCl-induced bioactive GA deficiency inhibits rice seed germination by decreasing α-amylase activity via down-regulation of α-amylase gene expression. Additionally, exogenous bioactive GA rescues NaCl-inhibited seed germination by enhancing α-amylase activity. Thus, NaCl treatment reduces bioactive GA content through promotion of bioactive GA inactivation, which in turn inhibits rice seed germination by decreasing α-amylase activity via down-regulation of α-amylase gene expression.

## Introduction

Soil salinity is an abiotic stress that adversely affects agricultural production throughout the world. It is estimated that approximately 6% of all land and 20% of irrigated land are affected by salinity (Munns and Tester, 2008). Additionally, the area of saline agricultural land is increasing annually, mainly due to irrigation (FAO, 2002). Rice (*Oryza sativa* L.), one of the most important food crops, is seriously affected by salinity (Munns and Tester, 2008). Salinity inhibits seed germination as well as seedling growth of rice (Anuradha and Rao, 2001), reduces photosynthesis, promotes senescence, and ultimately reduces production in rice (Tuteja et al., 2013).

Seed germination is a crucial phase in plant life that plays important roles in seedling establishment and subsequent growth (Bewley, 1997). Germination is regulated by multiple endogenous factors, such as plant hormones, and by environmental conditions, including temperature and light (Qu et al., 2008; Weitbrecht et al., 2011; Cho et al., 2012; Miransari and Smith, 2014). Salinity inhibits seed germination (Gill et al., 2003; Chang et al., 2010), whereas gibberellin (GA) promotes seed germination (Kaneko et al., 2002; Dong et al., 2012; Meng et al., 2016). Previously, we showed that rice seed germination is significantly inhibited by salinity, which can be alleviated by GA (Yin et al., 2009). However, the inhibitory mechanism by which salinity affects seed germination remains elusive. GA and abscisic acid (ABA) were recognized as the major hormones that have antagonistic effect on regulation of seed germination (Llanes et al., 2016; Shu et al., 2016). It has been reported that salinity inhibited soybean seed germination by decreasing the ratio of GA/ABA via decreased bioactive GA and increased ABA contents (Shu et al., 2017). However, the role of GA rather than ABA is important in regulating *Atriplex centralasiatica* and tomato seed germination under salt stress (Li et al., 2011; Nakaune et al., 2012).

Gibberellins are a group of tetracyclic diterpenoid phytohormones, and GA homeostasis plays important roles in regulating seed germination, plant growth and development (Mitsunaga and Yamaguchi, 1993; Richards et al., 2001). GA homeostasis is controlled by GA metabolism, including biosynthesis and inactivation (Hedden and Phillips, 2000; Frigerio et al., 2006). GA biosynthesis is catalyzed by enzymes of *ent*-copalyl diphosphate synthase (CPS), *ent*-kaurene synthase (KS), *ent*-kaurene oxidase (KO), *ent*-kaurene acid oxidase (KAO), GA 20-oxidase (GA20ox), and GA 3-oxidase (GA3ox). More than 100 GAs have been identified in plants (Macmillan, 2002); however, only a few, including GA_1_, GA_3_, GA_4_, and GA_7_, are the bioactive forms in higher plants, whereas others are precursors and inactivated products of bioactive GAs (Yamaguchi, 2008). In rice, GA_1_ and GA_4_ are the predominant bioactive GA forms (Kobayashi et al., 1988). Bioactive GAs can be inactivated by GA 2-oxidase (GA2ox) (Olszewski et al., 2002; Yamaguchi, 2008; Hedden and Thomas, 2012). The bioactive GA content and the germination rate are correlated with the expression levels of GA metabolic genes. A *germination-defective1* (*gd1*) mutant identified in rice was defective in seed gerrmination due to increased expression of *OsGA2ox3* and reduced expression of *OsGA20ox1*, *OsGA20ox2*, and *OsGA3ox2* (Guo et al., 2013). Loss-of-function mutation in *CPS* decreased bioactive GA content and inhibited seed germination (Lee et al., 2002). Rice seed germination of *OsGA2ox6*_ACT_ mutant with increased accumulation of *OsGA2ox6* mRNA was inhibited due to GA deficiency (Lo et al., 2008).

In cereal seeds, carbohydrates and proteins stored in the endosperm are mobilized during seed germination to provide energy and substrates for developing seedlings. During seed germination, bioactive GAs are synthesized in the embryo and transported to the aleurone layer to induce α-amylase gene expression and α-amylase synthesis. Then, α-amylase is secreted into the endosperm to hydrolyze the stored starch (Kaneko et al., 2002). α-amylase (EC 3.2.1.1) is the major enzyme involved in the hydrolysis of starch to glucose, and accounts for 40–60% of *de novo* protein synthesis in grains (Sun and Henson, 1991). Previous studies showed that seed germination was significantly inhibited by salinity but could be rescued by GA (Li et al., 2016; Shu et al., 2017). These results imply that salinity-inhibited seed germination may be caused by a decrease in GA content. However, the underlying mechanism of salinity-inhibited seed germination remains unclear. To elucidate the mechanism, the effects of salinity on GA metabolism, α-amylase gene expression, and α-amylase activity were investigated in this study.

## Materials and Methods

### Plant Materials and Germination Treatments

Indica rice Zhenshan 97 (*O. sativa* L.) seeds were used in this study. Rice Zhenshan 97 is an inbred variety that is widely used in China. The rice seeds were sterilized according to the method of Yin et al. (2011). The sterilized seeds were germinated in 9-cm Petri dishes with 35 mL distilled water (control), 120 mM NaCl, or 120 mM NaCl + 50 μM GA_3_. All seeds were germinated in an artificial climate incubator (HP 1500 GS) at 28°C for 3, 6, 12, 24, 48, 72, and 96 h.

### Seed Germination Analysis

Rice seeds were incubated in 9-cm Petri dishes with distilled water (control), 120 mM NaCl, or 120 mM NaCl + 50 μM GA_3_. The germination rates were calculated after 96 h incubation. Every treatment had five replicates, and each replicate included 50 seeds. A seed was recorded as germinated when the root length was ≥ 1 cm and the shoot length was ≥ 0.5 cm.

### Determination of Bioactive GA Content

Rice seeds were incubated in 9-cm Petri dishes with distilled water (control) or 120 mM NaCl. After 96 h incubation, the embryos from germinating seeds were collected for GA measurements. Quantification of endogenous GAs was performed as described (Chen et al., 2012).

### Quantitative Assay for α-Amylase Activity

Rice seeds were sterilized according to the method of Yin et al. (2011) and then incubated with distilled water (control), 120 mM NaCl, or 120 mM NaCl + 50 μM GA_3_ at 28°C. After 24, 48, 72, and 96 h incubation, the crude extract was prepared according to the method of Sottirattanapan et al. (2017). Each sample consisting of 15 germinating seeds was collected, ground, and mixed with 100 mL chilled distilled water for enzyme extraction. The mixture was soaked in a cooling bath at 4°C for 10 min with occasional agitation. After soaking, the mass was squeezed through a nylon cloth to collect the extract. The extract was then centrifuged at 10,000 × *g* for 10 min at 4°C, and the clear supernatant was used as the crude extract.

α-Amylase activity was quantitatively assayed by a slightly modified version of the 3,5-dinitrosalicylic acid method of Miller (1959). The crude enzyme extract was heated for 15 min at 70°C. Then, 1 mL of the crude enzyme extract was mixed with 1 mL of 1% soluble starch dissolved in sodium acetate buffer at pH 5.6. The mixture was incubated for 15 min at 40°C and then boiled for 5 min in the presence of 2 mL of 3,5-dinitrosalicylic acid. The amount of released reducing sugar was measured using a UV-vis spectrophotometer (UV-2100, Unico Instrument Co., Ltd., Shanghai, China) at 540 nm with maltose as the reducing sugar standard. One unit of α-amylase activity was defined as the amount of enzyme that produced 1 μM of maltose per minute under the enzyme activity conditions.

### Qualitative Assay for α-Amylase Activity

To qualitatively assess the effect of salinity on α-amylase activity, we used a starch plate test using embryoless half-seeds according to the method of Xie et al. (2007). Rice seeds were sterilized according to the method of Yin et al. (2011), and then incubated with distilled water (control), 120 mM NaCl, or 120 mM NaCl + 50 μM GA_3_ at 28°C for 48 h. The seeds were then cut transversely to remove the embryos, and the embryoless half-seeds were then placed on 2% agar in 9-cm Petri dishes with the cut edge on the agar. The agar plates included 0.2% soluble potato starch, 20 mM CaCl_2_, 20 mM sodium succinate pH 5.0, and one of the following treatments: control (without NaCl and GA), 120 mM NaCl, or 120 mM NaCl + 50 μM GA_3_. The dishes were incubated at 28°C for 48 h. After incubation, the plates were flooded with I_2_/KI solution (2.8 mM I_2_ + 43.4 mM KI in 0.2 N HCl). After 5 min, the reaction between starch and iodine turned the agar plates blue-purple. The agar around the half-seeds with α-amylase activity remained colorless due to the hydrolysis of starch by α-amylase. The colorless area increased as the α-amylase activity increased.

### RNA Isolation, cDNA Synthesis, and Quantitative Real-Time PCR (qRT-PCR) Analysis

Rice seeds were incubated in 9-cm Petri dishes with distilled water (control), 120 mM NaCl, or 120 mM NaCl + 50 μM GA_3_. After 3, 6, 12, 24, 48, 72, and 96 h incubation, total RNA from the seed embryos was extracted using an RNAprep Pure Plant kit (Tiangen Biotech, Beijing, China) according to the manufacturer’s instructions, and the total RNA from embryoless half-seeds was extracted using the method of Ismail et al. (2009). Additionally, 1.5 μg of total RNA was used for first-strand cDNA synthesis using a FastQuant RT kit (Tiangen Biotech, Beijing, China) according to the manufacturer’s instructions.

Quantitative Real-Time PCR was performed using 2 × HSYBR qPCR mix (Zoman Biotech, Beijing, China) on a qTower 2.2 real-time PCR system (Analytik Jena, Jena, Germany) according to the manufacturer’s instructions. Each analysis had three biological repeats with three technical replicates. The comparative threshold (CT) method was applied to calculate relative gene expression, and rice *OsACTIN* gene expression was used as an internal control to normalize expression of the target genes. Supplementary Table 1 lists the gene-specific primers used for qRT-PCR.

### Accession Numbers

The GenBank accession numbers of the genes examined by qRT-PCR are: *OsCPS1* (LOC_Os02g17780), *OsKS1* (LOC_Os04g52230), *OsKO1* (LOC_Os06g37330), *OsKAO* (LOC_Os06g 02019), *OsGA20ox1* (LOC_Os03g63970), *OsGA3ox2* (LOC_ Os01g08220), *OsGA2ox1* (LOC_Os05g06670), *OsGA2ox2* (LOC_Os01g22910), *OsGA2ox3* (LOC_Os01g55240), *OsGA2ox5* (LOC_Os07g01340), *OsGA2ox6* (LOC_Os04g44150), *OsGA2ox9* (LOC_Os02g41954), *OsAmy1A* (LOC_Os02g52710), *OsAmy1C* (LOC_Os02g5270), *OsAmy3C* (LOC_Os09g28420), *OsAmy3E* (LOC_Os08g36900), and *OsACTIN* (LOC_Os03g50885).

### Statistical Analysis

Statistical analysis was performed using an independent-samples *t*-test, or one-way analysis of variance (ANOVA) followed by Duncan’s multiple range test with at least three replicates. *P* values < 0.05 were considered statistically significant. All data are expressed as means ± standard error (SE).

## Results

### Salinity-Inhibited Rice Seed Germination Was Correlated With Bioactive GA Deficiency

**FIGURE 1:**
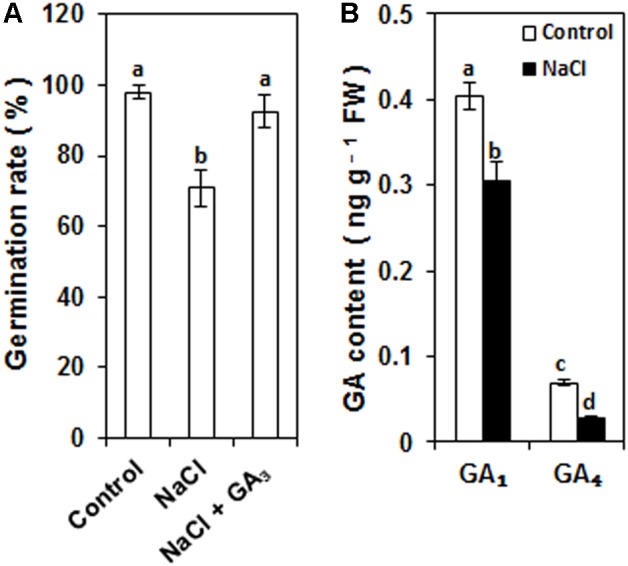
Effects of NaCl on the rice seed germination rate and the contents of GA_1_ and GA_4_ in germinating seeds. **(A)** Effects of NaCl and NaCl + GA_3_ on the rice seed germination rate. Rice seeds were incubated in 9-cm Petri dishes with distilled water (control), 120 mM NaCl, or 120 mM NaCl + 50 μM GA_3_. After 96 h of incubation, the seed germination rate was calculated from five biological replicates. **(B)** Effects of NaCl on GA_1_ and GA_4_ contents in germinating seeds. Rice seeds were incubated in 9-cm Petri dishes with distilled water (control) or 120 mM NaCl. After 96 h incubation, the GA_1_ and GA_4_ contents of seed embryos were detected with three biological replicates. Data are presented as means ± SE. Significant differences (*P* < 0.05) are indicated by different letters.

After 96 h incubation, the seed germination rate of control seeds was about 98% (**Figure 1A**). NaCl treatment significantly inhibited seed germination, and the seed germination rate of NaCl-treated seeds was 71% (**Figure 1A**). However, the decrease in the seed germination rate was rescued by GA_3_ application (**Figure 1A**). This result implies that the decrease in the seed germination rate may have been caused by a decrease in the GA content, which would explain why the decrease in the seed germination rate was rescued by GA_3_ application.

GA_1_ and GA_4_ are the major bioactive GA forms in rice. To examine whether the bioactive GA content was reduced by salinity, the amounts of GA_1_ and GA_4_ in seed embryos were determined after 96 h incubation. As shown in **Figure 1B**, the amounts of both GA_1_ and GA_4_ were decreased by NaCl treatment compared to the control. GA_1_ and GA_4_ contents decreased by 24% and 60%, respectively. This result demonstrates that salinity significantly decreased the bioactive GA content of germinating seeds.

### Effect of Salinity on Bioactive GA Metabolism

Bioactive GAs are cooperatively regulated by biosynthesis and inactivation. To investigate how the bioactive GA content was decreased by NaCl treatment, the effect of NaCl treatment on the expression of GA biosynthetic and inactivated genes was investigated during seed germination.

**FIGURE 2:**
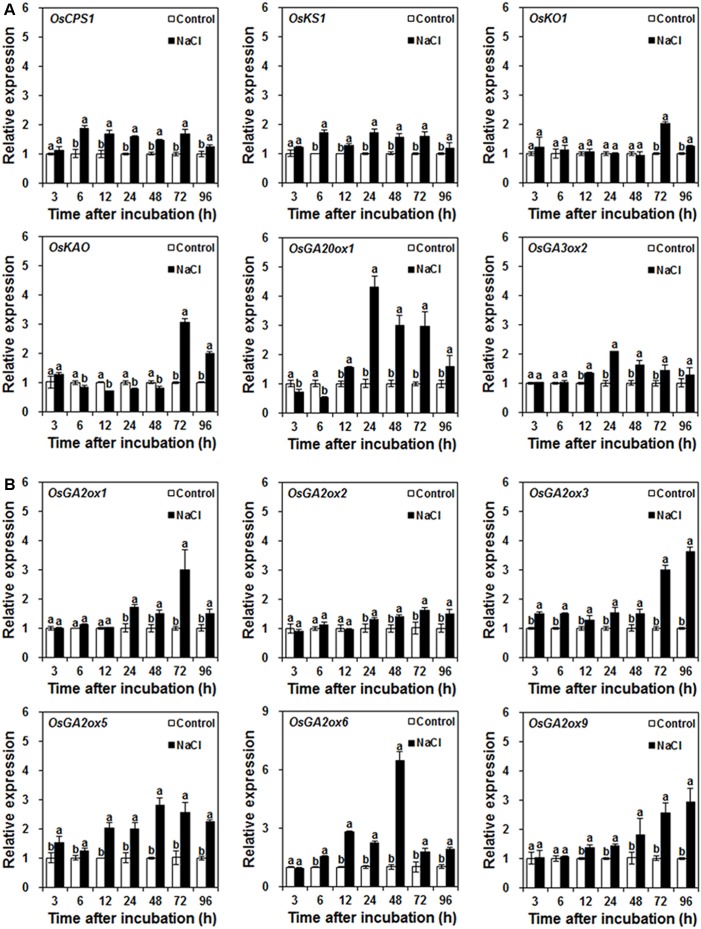
Effects of control and NaCl on GA metabolic gene expression during rice seed germination. **(A)** Effects of control and NaCl on GA biosynthetic gene expression. **(B)** Effects of control and NaCl on GA-inactivated gene expression. Rice seeds were incubated in 9-cm Petri dishes with distilled water (control) or 120 mM NaCl. After 3, 6, 12, 24, 48, 72, and 96 h incubation, GA metabolic gene expression was analyzed. Data are presented as means ± SE. Three biological replicates with three technical replicates were included in statistical analysis and error range analysis. The expression levels of GA metabolic genes in control were set as 1. Different letters indicate significantly different (*P* < 0.05) gene expression in control seed embryos compared to NaCl-treated seed embryos at the same time.

The temporal expression profiles of GA biosynthetic genes showed that NaCl treatment resulted in up-regulation in the expression levels of *OsCPS1* and *OsKS1* from 6 to 96 h after incubation, of *OsGA3ox2* from 12 to 96 h after incubation, and of *OsKO1* from 72 to 96 h after incubation (**Figure 2A**). Although NaCl treatment slightly down-regulated *OsKAO* expression from 6 to 48 h after incubation and moderately down-regulated *OsGA20ox1* expression from 3 to 6 h after incubation, NaCl treatment significantly up-regulated the expression levels of *OsGA20ox1* from 12 to 96 h after incubation and of *OsKAO* from 72 to 96 h after incubation (**Figure 2A**).

Six *GA2oxs*, including *OsGA2ox1*, *OsGA2ox2*, *OsGA2ox3*, *OsGA2ox5*, *OsGA2ox6*, and *OsGA2ox9*, are responsible for regulating rice seed germination (Lo et al., 2008). As shown in **Figure 2B**, the expression levels of six *OsGA2oxs* were up-regulated by NaCl treatment during different time periods during rice seed germination. *OsGA2ox3* and *OsGA2ox5* responded quickly to NaCl treatment; they were up-regulated within 3 h after incubation; thereafter, their expression levels were higher than that of the control. *OsGA2ox6* expression was up-regulated 6 h after incubation; from then on, it was also higher than that of the control. *OsGA2ox9* expression was up-regulated by NaCl treatment from 12 to 96 h after incubation. In contrast, *OsGA2ox1* and *OsGA2ox2* responded slowly to NaCl treatment and were up-regulated after 24 h incubation.

### Salinity Decreased α-Amylase Activity

**FIGURE 3:**
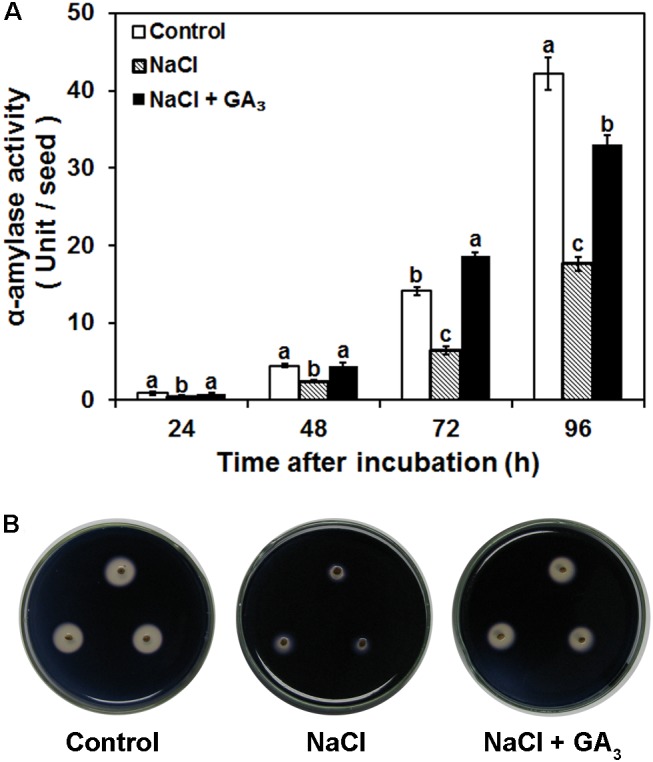
Effects of control, NaCl, and NaCl + GA_3_ on α-amylase activity during rice seed germination. **(A)** Quantitative differences in α-amylase activity between different treatments. Rice seeds were sterilized and then incubated with different solutions at 28°C for 24, 48, 72, and 96 h. Quantitative α-amylase activity was assayed by the 3,5-dinitrosalicylic acid method. Each value was calculated from five biological replicates. Data are presented as means ± SE. Significant differences (*P* < 0.05) in α-amylase activity between different treatments are indicated by different letters at the same time. **(B)** Qualitative differences in α-amylase activity between different treatments. Three biological replicates with three technical replicates were included for each qualitative α-amylase activity assay.

Starch is the most abundant reserve in rice seeds. α-Amylase is a crucial enzyme that participates in the degradation of starch granules into small organic molecules to provide energy and nutrients for seed germination. To test whether salinity-induced seed germination inhibition was mediated by a decrease in α-amylase activity, the effects of distilled water (control), 120 mM NaCl, and 120 mM NaCl + 50 μM GA_3_ on α-amylase activity were investigated. The quantitative data demonstrated that α-amylase activity was significantly decreased by NaCl treatment compared to the control. Specifically, NaCl treatment reduced α-amylase activity by 49, 45, 54, and 58% after 24, 48, 72, and 96 h incubation, respectively (**Figure 3A**). The NaCl-induced decrease in α-amylase activity from 24 to 96 h was alleviated by GA_3_ application (**Figure 3A**). The same result was also demonstrated by the qualitative data (**Figure 3B**). The colorless areas around embryoless half-seeds treated with NaCl were much smaller than those of the control, and the decrease in the colorless areas was also rescued by GA_3_ application (**Figure 3B**). The agar surrounding half-seeds with α-amylase activity remained colorless due to starch hydrolysis by α-amylase. The colorless area increased as the α-amylase activity increased. Thus, the qualitative results also indicate that α-amylase activity was significantly decreased by NaCl treatment, and this NaCl-induced decrease in α-amylase activity was rescued by GA_3_ application.

### Salinity Down-Regulated α-Amylase Gene Expression

**FIGURE 4:**
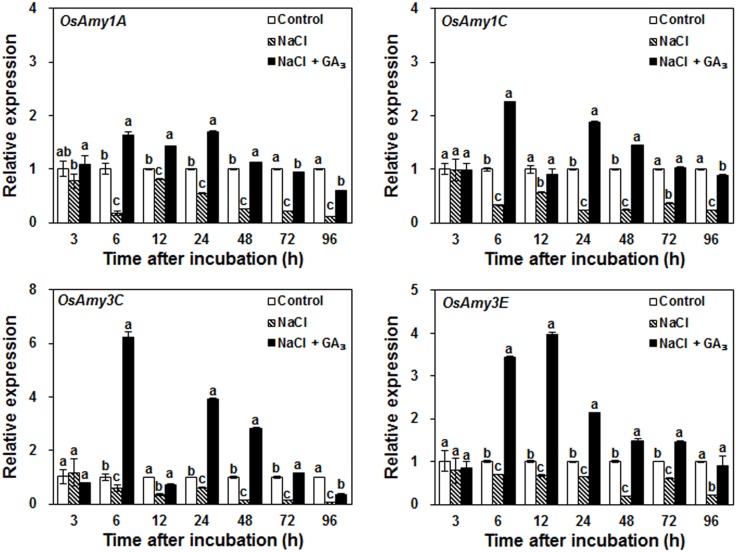
Effects of control, NaCl, and NaCl + GA_3_ on α-amylase gene expression during rice seed germination. Rice seeds were incubated in 9-cm Petri dishes with distilled water (control), 120 mM NaCl, or 120 mM NaCl + 50 μM GA_3_. After 3, 6, 12, 24, 48, 72, and 96 h incubation, embryoless half-seeds were harvested for gene expression analysis. Data are presented as means ± SE. Three biological replicates with three technical replicates were included for statistical analysis and error range analysis. The expression levels of α-amylase genes in control were set as 1. Different letters indicate significantly different (*P* < 0.05) gene expression in different treatments at the same time.

α-Amylases, such as OsAmy1A (RAmy1A), OsAmy1C (RAmy1C), OsAmy3C (RAmy3C), and OsAmy3E (RAmy3E), are required for starch degradation during seed germination (Huang et al., 1990; Karrer et al., 1991; Sutliff et al., 1991). To explore how salinity may decrease α-amylase activity, the effects of the control, NaCl, and NaCl + GA_3_ treatments on α-amylase gene expression, which is closely related to the production of α-amylase, were analyzed. The results show that at 6 h after incubation, the expression levels of all α-amylase genes were significantly decreased by NaCl treatment, whereas exogenous application of GA_3_ rescued this decrease (**Figure 4**). This result indicates that salinity decreases α-amylase activity mainly via down-regulation of α-amylase gene expression.

## Discussion

### Salinity Inhibits Seed Germination by Decreasing Bioactive GA Content

Gibberellins play a critical role in promoting seed germination. It has been reported that GA biosynthesis is induced during seed germination (Ayele et al., 2012). GA biosynthetic inhibitors suppress seed germination, and exogenous GA reverses the inhibitory effect (Gallardo et al., 2002). GA-deficient mutants, such as *ga1-3* in *Arabidopsis* and *gib-1* in tomato, are difficult to germinate without exogenous GA (Ni and Bradford, 1993; Lee et al., 2002).

Our results demonstrate that NaCl treatment inhibits rice seed germination. Furthermore, the NaCl-inhibited seed germination can be rescued by exogenous GA_3_ application (**Figure 1A**). These results imply that NaCl may inhibit seed germination by decreasing the bioactive GA content. In rice, GA_1_ and GA_4_ are the predominant bioactive GA forms (Kobayashi et al., 1988). Thus, to test this, the effects of control and NaCl treatments on GA_1_ and GA_4_ contents were analyzed. Compared to control treatment, NaCl treatment significantly decreased the GA_1_ and GA_4_ contents (**Figure 1B**). These results indicate that salinity inhibits rice seed germination by decreasing the bioactive GA content. Although salinity-induced bioactive GA deficiency has been reported in rice previously, the forms of bioactive GA cannot be distinguished in the previous study (Kim et al., 2006). In this study, our data demonstrated that during rice seed germination, the content of GA_1_ was much higher than that of GA_4_, and the salinity-decreased content (0.1 ng g^-1^ FW) of GA_1_ was much more than that (0.04 ng g^-1^ FW) of GA_4_ (**Figure 1B**). Our data suggest that GA_1_ is the predominant form of bioactive GA during rice seed germination, and salinity-induced bioactive GA deficiency is mainly due to decreased GA_1_ content. In contrast, during *Suaeda salsa* seed germination, GA_4_ was regarded as the main form of bioactive GA, and salinity decreased GA_4_ content during seed germination (Li et al., 2016).

Besides GA, ABA also plays an important role in regulating seed germination. It was recognized that GA and ABA antagonistically regulate seed germination (Li et al., 2016; Shu et al., 2016), and NaCl inhibited soybean seed germination by decreasing the ratio of GA/ABA via decreased bioactive GA and increased ABA contents (Shu et al., 2017). On the contrary, in *Atriplex centralasiatica* seeds under saline conditions, brown seeds contained more active GAs than black seeds, although they contained a similar content of ABA, and the germination rate of brown seeds was higher than that of black seeds under the same salt stress (Li et al., 2011). Moreover, the ABA contents in the control (distilled water-primed) tomato seeds and NaCl-primed tomato seeds were not significantly different both during and after the priming treatments (Nakaune et al., 2012). These results suggested that the role of GA rather than ABA is important in regulating *Atriplex centralasiatica* and tomato seed germination under salt stress. However, whether and how ABA affects rice seed germination under salt stress is largely unknown so far. More research on the effect of ABA on rice seed germination under salt stress is needed in future.

### Salinity Decreases Bioactive GA Content by Enhancing Bioactive GA Inactivation

Bioactive GA contents are cooperatively regulated by biosynthesis and inactivation. In rice, CPS, KS, KO, KAO, GA20ox, and GA3ox catalyze GA biosynthesis, and GA2ox can inactivate bioactive GAs (Olszewski et al., 2002; Yamaguchi, 2008; Hedden and Thomas, 2012). To explore the mechanism by which NaCl decreases the bioactive GA content, the effects of NaCl treatment on the expression of bioactive GA biosynthetic and inactivated genes were investigated.

Our results demonstrate that, during rice seed germination, GA biosynthetic genes were up-regulated by NaCl treatment in the majority of the observed time courses (**Figure 2A**). Additionally, all *GA2ox* genes, which are responsible for regulating rice seed germination (Lo et al., 2008), were also up-regulated by NaCl treatment (**Figure 2B**). These results, in addition to the evidence that the bioactive GA content was decreased by NaCl treatment, indicate that NaCl induces bioactive GA deficiency by enhancing bioactive GA inactivation rather than by inhibiting bioactive GA biosynthesis; they also imply that up-regulated GA biosynthetic gene expression may be a consequence of negative feedback regulation of NaCl-induced bioactive GA deficiency. Our results are supported in *Arabidopsis*, a dicotyledonous model plant that also responds to high-salinity stress through a decrease in endogenous GA content and up-regulation of *GA2ox* genes expression (Magome et al., 2008). Our results are also consistent with a previous report that expression of GA biosynthetic genes, such as *GA20ox* and *GA3ox*, was negatively regulated by GA content (Carrera et al., 1999; Rieu et al., 2008; Huang et al., 2010). In contrast, during soybean seed germination, salinity decreased bioactive GA content by negatively regulating GA biosynthesis (Shu et al., 2017).

### Salinity-Induced GA Deficiency Inhibits Seed Germination by Decreasing α-Amylase Activity

In cereals, seed germination is dependent on the degradation of storage reserves in mature seeds, and the sugars from starch hydrolysis are the major source of energy for seedling emergence (Beck and Ziegler, 1989). α-Amylase is the major enzyme involved in starch mobilization; thus, α-amylase activity is an important factor in seed germination (Karrer et al., 1993). In this study, to test whether NaCl-induced bioactive GA deficiency inhibited seed germination by decreasing α-amylase activity, the effects of control, NaCl, and NaCl + GA_3_ treatments on α-amylase activity and the rate of seed germination were investigated.

Our results demonstrate that NaCl treatment significantly decreases α-amylase activity and the seed germination rate, and these effects can be rescued by exogenous GA_3_ during rice seed germination (**Figures 1A**, **3**). Furthermore, we found positive relationships between bioactive GA content and α-amylase activity and between α-amylase activity and the rice seed germination rate (**Figures 1**, **3A**). These results suggest that NaCl-induced bioactive GA deficiency inhibits seed germination by decreasing α-amylase activity. This is supported by a previous report that α-amylase activity was lower in GA-deficient dwarf rice, such as Tan-ginbozu (*dx* mutant), Waito-C (*dy* mutant), and Kotake-tamanishiki, than in the normal rice Nipponbare (Mitsunaga and Yamaguchi, 1993). Additionally, uniconazole (an inhibitor of GA biosynthesis) treatment inhibits α-amylase production in rice seed, whereas GA_3_ application reverses the inhibitory effect of uniconazole (Mitsunaga and Yamaguchi, 1993). GA_3_ application can also reverse the inhibitory effect of NaCl on amylase activity in other germinating seeds, such as *Amaranthus caudatus* and chickpea (*Cicer arietinum* L.) seeds (Kaur et al., 1998; Bialecka and Kpczynski, 2009).

### Salinity-Induced GA Deficiency Decreases α-Amylase Activity by Down-Regulating α-Amylase Gene Expression

It has been reported that rice α-amylase genes, such as *OsAmy1A*, *OsAmy1C*, *OsAmy3C*, and *OsAmy3E*, were significantly up-regulated by GA in wild-type seeds but not in the GA receptor mutant *gid1* (a null mutant for GID1). In GA signaling repressor mutant *slr1* (a null mutant for DELLA) seeds, regardless of GA treatment, α-amylase gene expression levels were similar to those of GA-treated wild-type seeds (Yano et al., 2015). These previous results confirm that GA regulates α-amylase activity through transcriptional regulation in rice. Our results indicate that NaCl treatment significantly decreases the bioactive GA content, which, in turn, down-regulates α-amylase gene expression and enzymatic activity; these down-regulated parameters can be rescued by GA_3_ application (**Figures 1B**, **3**, **4**). Similarly, a decline in GA content was accompanied by decreased transcription of *α-Amy1* and *α-Amy2* gene in wheat grain due to ectopic expression of *PcGA2ox1* (Appleford et al., 2007). The results of our current study and previous research indicate that NaCl-induced GA deficiency decreases α-amylase activity by down-regulating the expression of α-amylase genes.

## Conclusion

In this study, we provided evidence that salinity inhibits rice seed germination by decreasing the bioactive GA content, as a result of an increase in bioactive GA inactivation. Furthermore, bioactive GA deficiency inhibits seed germination by decreasing α-amylase activity via down-regulation of α-amylase gene expression.

## Author Contributions

LL and WX performed most of the experiments. HL, BW, and SH were involved in the experiments. LL and CY analyzed the data. CY and HZ designed the experiments. LL and CY wrote the manuscript. All the authors reviewed the manuscript and recommended its submission.

## Conflict of Interest Statement

The authors declare that the research was conducted in the absence of any commercial or financial relationships that could be construed as a potential conflict of interest.

## References

[B1] Anuradha S. Rao S. S. R. (2001). Effect of brassinosteroids on salinity stress induced inhibition of seed germination and seedling growth of rice (*Oryza sativa L.*). *Plant Growth Regul.* 33 151–153. 10.1023/a:1017590108484

[B2] Appleford N. E. Wilkinson M. D. Ma Q. Evans D. J. Stone M. C. Pearce S. P. (2007). Decreased shoot stature and grain alpha-amylase activity following ectopic expression of a gibberellin 2-oxidase gene in transgenic wheat. *J. Exp. Bot.* 58 3213–3226. 10.1093/jxb/erm166 17916639

[B3] Ayele B. T. Ozga J. A. Wickramarathna A. D. Reinecke D. M. (2012). Gibberellin metabolism and transport during germination and young seedling growth of pea (*Pisum sativum* L.). *J. Plant Growth Regul.* 31 235–252. 10.1007/s00344-011-9234-8

[B4] Beck E. Ziegler P. (1989). Biosynthesis and degradation of starch in higher plants. *Annu. Rev. Plant Physiol. Plant Mol. Biol.* 40 95–117. 10.1146/annurev.arplant.40.1.95

[B5] Bewley J. D. (1997). Seed germination and dormancy. *Plant Cell* 9 1055–1066. 10.1105/tpc.9.7.1055 12237375 PMC156979

[B6] Bialecka B. Kpczynski J. (2009). Effect of ethephon and gibberellin A3 on *Amaranthus caudatus* seed germination and α- and β-amylase activity under salinity stress. *Acta Biol. Cracov. Bot.* 51 119–125.

[B7] Carrera E. Jackson S. D. Prat S. (1999). Feedback control and diurnal regulation of gibberellin 20-oxidase transcript levels in potato. *Plant Physiol.* 119 765–773. 10.1104/pp.119.2.765 9952473 PMC32154

[B8] Chang C. Wang B. Shi L. Li Y. Duo L. Zhang W. (2010). Alleviation of salt stress-induced inhibition of seed germination in cucumber (*Cucumis sativus* L.) by ethylene and glutamate. *J. Plant Physiol.* 167 1152–1156. 10.1016/j.jplph.2010.03.018 20605252

[B9] Chen M. L. Fu X. M. Liu J. Q. Ye T. T. Hou S. Y. Huang Y. Q. (2012). Highly sensitive and quantitative profiling of acidic phytohormones using derivatization approach coupled with nano-LC-ESI-Q-TOF-MS analysis. *J. Chromatogr. B* 905 67–74. 10.1016/j.jchromb.2012.08.005 22917596

[B10] Cho J. N. Ryu J. Y. Jeong Y. M. Park J. Song J. J. Amasino R. M. (2012). Control of seed germination by light-induced histone arginine demethylation activity. *Dev. Cell* 22 736–748. 10.1016/j.devcel.2012.01.024 22483719

[B11] Dong T. Tong J. Xiao L. Cheng H. Song S. (2012). Nitrate, abscisic acid and gibberellin interactions on the thermoinhibition of lettuce seed germination. *Plant Growth Regul.* 66 191–202. 10.1007/s10725-011-9643-5

[B12] FAO (2002). *Crops and Drops: Making the Best use of Water for Agriculture. FAO, Rome*. Available at http://www.fao.org/docrep/w5146e/w5146e0a.htm

[B13] Frigerio M. Alabadí D. Pérezgómez J. Garcíacárcel L. Phillips A. L. Hedden P. (2006). Transcriptional regulation of gibberellin metabolism genes by auxin signaling in Arabidopsis. *Plant Physiol.* 142 553–563. 10.1104/pp.106.084871 16905669 PMC1586059

[B14] Gallardo K. Job C. Groot S. P. C. Puype M. Demol H. Vandekerckhove J. (2002). Proteomics of Arabidopsis seed germination. A comparative study of wild-type and gibberellin-deficient seeds. *Plant Physiol.* 129 823–837. 10.1104/pp.002816 12068122 PMC161704

[B15] Gill P. K. Sharma A. D. Singh P. Bhullar S. S. (2003). Changes in germination, growth and soluble sugar contents of *Sorghum bicolor* (L.) Moench seeds under various abiotic stresses. *Plant Growth Regul.* 40 157–162. 10.1023/A:1024252222376

[B16] Guo X. Hou X. Fang J. Wei P. Bo X. Chen M. (2013). The rice *GERMINATION DEFECTIVE 1*, encoding a B3 domain transcriptional repressor, regulates seed germination and seedling development by integrating GA and carbohydrate metabolism. *Plant J.* 75 403–416. 10.1111/tpj.12209 23581288 PMC3813988

[B17] Hedden P. Phillips A. L. (2000). Gibberellin metabolism: new insights revealed by the genes. *Trends Plant Sci.* 5 523–530. 10.1016/S1360-1385(00)01790-8 11120474

[B18] Hedden P. Thomas S. G. (2012). Gibberellin biosynthesis and its regulation. *Biochem. J.* 444 11–25. 10.1042/BJ20120245 22533671

[B19] Huang J. Tang D. Shen Y. Qin B. Hong L. You A. (2010). Activation of gibberellin 2-oxidase 6 decreases active gibberellin levels and creates a dominant semi-dwarf phenotype in rice (*Oryza sativa* L.). *J. Genet. Genomics* 37 23–36. 10.1016/S1673-8527(09)60022-9 20171575

[B20] Huang N. Koizumi N. Reinl S. Rodriguez R. L. (1990). Structural organization and differential expression of rice α-amylase genes. *Nucleic Acids Res.* 18 7007–7014. 10.1093/nar/18.23.7007 2263460 PMC332763

[B21] Ismail A. M. Ella E. S. Vergara G. V. Mackill D. J. (2009). Mechanisms associated with tolerance to flooding during germination and early seedling growth in rice (*Oryza sativa*). *Ann. Bot.* 103 197–209. 10.1093/aob/mcn211 19001425 PMC2707318

[B22] Kaneko M. Itoh H. Ueguchi-Tanaka M. Ashikari M. Matsuoka M. (2002). The α-amylase induction in endosperm during rice seed germination is caused by gibberellin synthesized in epithelium. *Plant Physiol.* 128 1264–1270. 10.1104/pp.010785 11950975 PMC154254

[B23] Karrer E. E. Chandler J. M. Foolad M. R. Rodriguez R. L. (1993). Correlation between α-amylase gene expression and seedling vigor in rice. *Euphytica* 66 163–169. 10.1007/BF00025299

[B24] Karrer E. E. Litts J. C. Rodriguez R. L. (1991). Differential expression of α-amylase genes in germinating rice and barley seeds. *Plant Mol. Biol.* 16 797–805. 10.1007/BF00015072 1859866

[B25] Kaur S. Gupta A. K. Kaur N. (1998). Gibberellin A3 reverses the effect of salt stress in chickpea (*Cicer arietinum* L.) seedlings by enhancing amylase activity and mobilization of starch in cotyledons. *Plant Growth Regul.* 26 85–90. 10.1023/A:1006008711079

[B26] Kim S. K. Son T. K. Park S. Y. Lee I. J. Lee B. H. Kim H. Y. (2006). Influences of gibberellin and auxin on endogenous plant hormone and starch mobilization during rice seed germination under salt stress. *J. Environ. Biol.* 27 181–186.

[B27] Kobayashi M. Yamaguchi I. Murofushi N. Ota Y. Takahashi N. (1988). Fluctuation and localization of endogenous gibberellins in rice. *Agric. Biol. Chem.* 52 1189–1194. 10.1080/00021369.1988.10868799

[B28] Lee S. Cheng H. King K. E. Wang W. He Y. Hussain A. (2002). Gibberellin regulates *Arabidopsis* seed germination via *RGL2*, a *GAI/RGA*-like gene whose expression is up-regulated following imbibition. *Genes Dev.* 16 646–658. 10.1101/gad.969002 11877383 PMC155355

[B29] Li W. Liu X. Hanada A. Khan M. A. (2011). Effect of cold stratification, scarification and hormones on germination of dimorphic seeds of *Atriplex centralasiatica* under saline conditions. *Seed Sci. Technol.* 39 82–92. 10.15258/sst.2011.39.1.08

[B30] Li W. Yamaguchi S. Khan M. A. An P. Liu X. Tran L. S. P. (2016). Roles of gibberellins and abscisic acid in regulating germination of *Suaeda salsa* dimorphic seeds under salt stress. *Front. Plant Sci.* 6:1235. 10.3389/fpls.2015.01235 26793214 PMC4710750

[B31] Llanes A. Andrade A. Masciarelli O. Alemano S. Luna V. (2016). Drought and salinity alter endogenous hormonal profiles at the seed germination phase. *Seed Sci. Res.* 26 1–13. 10.1017/S0960258515000331

[B32] Lo S. F. Yang S. Y. Chen K. T. Hsing Y. I. Zeevaart J. A. Chen L. J. (2008). A novel class of gibberellin 2-oxidases control semidwarfism, tillering, and root development in rice. *Plant Cell* 20 2603–2618. 10.1105/tpc.108.060913 18952778 PMC2590730

[B33] Macmillan J. (2002). Occurrence of gibberellins in vascular plants, fungi, and bacteria. *J. Plant Growth Regul.* 20 387–442. 10.1007/s003440010038 11986764

[B34] Magome H. Yamaguchi S. Hanada A. Kamiya Y. Oda K. (2008). The DDF1 transcriptional activator upregulates expression of a gibberellin-deactivating gene, *GA2ox7*, under high-salinity stress in Arabidopsis. *Plant J.* 56 613–626. 10.1111/j.1365-313X.2008.03627.x 18643985

[B35] Meng Y. Chen F. Shuai H. Luo X. Ding J. Tang S. (2016). Karrikins delay soybean seed germination by mediating abscisic acid and gibberellin biogenesis under shaded conditions. *Sci. Rep.* 6:22073. 10.1038/srep22073 26902640 PMC4763256

[B36] Miller G. L. (1959). Use of dinitrosalicylic acid reagent for determination of reducing sugar. *Anal. Chem.* 31 426–428. 10.1021/ac60147a030

[B37] Miransari M. Smith D. L. (2014). Plant hormones and seed germination. *Environ. Exp. Bot.* 99 110–121. 10.1016/j.envexpbot.2013.11.005

[B38] Mitsunaga S. Yamaguchi J. (1993). Induction of α-amylase is repressed by uniconazole, an inhibitor of the biosynthesis of gibberellin, in a dwarf mutant of rice, waito-c. *Plant Cell Physiol.* 34 243–249. 10.1093/oxfordjournals.pcp.a078413

[B39] Munns R. Tester M. (2008). Mechanisms of salinity tolerance. *Annu. Rev. Plant Biol.* 59 651–681. 10.1146/annurev.arplant.59.032607.092911 18444910

[B40] Nakaune M. Hanada A. Yin Y. G. Matsukura C. Yamaguchi S. Ezura H. (2012). Molecular and physiological dissection of enhanced seed germination using short-term low-concentration salt seed priming in tomato. *Plant Physiol. Biochem.* 52 28–37. 10.1016/j.plaphy.2011.11.005 22305065

[B41] Ni B. R. Bradford K. J. (1993). Germination and dormancy of abscisic acid and gibberellin-deficient mutant tomato (*Lycopersicon esculentum*) seeds. *Plant Physiol.* 101 607–617. 10.1104/pp.101.2.607 12231716 PMC160610

[B42] Olszewski N. Sun T. P. Gubler F. (2002). Gibberellin signaling: biosynthesis, catabolism, and response pathways. *Plant Cell* 14(Suppl.), S61–S80. 10.1105/tpc.010476 12045270 PMC151248

[B43] Qu X. X. Huang Z. Y. Baskin J. M. Baskin C. C. (2008). Effect of temperature, light and salinity on seed germination and radicle growth of the geographically widespread halophyte shrub *Halocnemum strobilaceum*. *Ann. Bot.* 101 293–299. 10.1093/aob/mcm047 17428834 PMC2711011

[B44] Richards D. E. King K. E. Tahar Aitali A. Harberd N. P. (2001). How gibberellin regulates plant growth and development: a molecular genetic analysis of gibberellin signaling. *Annu. Rev. Plant Physiol. Plant Mol. Biol.* 52 67–88. 10.1146/annurev.arplant.52.1.67 11337392

[B45] Rieu I. Ruiz-Rivero O. Fernandez-Garcia N. Griffiths J. Powers S. J. Gong F. (2008). The gibberellin biosynthetic genes *AtGA20ox1* and *AtGA20ox2* act, partially redundantly, to promote growth and development throughout the Arabidopsis life cycle. *Plant J.* 53 488–504. 10.1111/j.1365-313X.2007.03356.x 18069939

[B46] Shu K. Liu X. D. Xie Q. He Z. H. (2016). Two faces of one seed: hormonal regulation of dormancy and germination. *Mol. Plant* 9 34–45. 10.1016/j.molp.2015.08.010 26343970

[B47] Shu K. Qi Y. Chen F. Meng Y. Luo X. Shuai H. (2017). Salt stress represses soybean seed germination by negatively regulating GA biosynthesis while positively mediating ABA biosynthesis. *Front. Plant Sci.* 8:1372. 10.3389/fpls.2017.01372 28848576 PMC5554363

[B48] Sottirattanapan P. Nantachai K. Daduang S. Funahashi T. Yamada M. (2017). Purification and characterization of amylase from roots of *Paederia foetida* Linn. *Biocatal. Agri. Biotechnol.* 10 329–335. 10.1016/j.bcab.2017.04.012

[B49] Sun Z. Henson C. A. (1991). A quantitative assessment of the importance of barley seed α-amylase, β-amylase, debranching enzyme, and α-glucosidase in starch degradation. *Arch. Biochem. Biophys.* 284 298–305. 10.1016/0003-9861(91)90299-X 1824915

[B50] Sutliff T. D. Huang N. Litts J. C. Rodriguez R. L. (1991). Characterization of an alpha-amylase multigene cluster in rice. *Plant Mol. Biol.* 16 579–591. 10.1007/BF00023423 1714318

[B51] Tuteja N. Sahoo R. K. Garg B. Tuteja R. (2013). OsSUV3 dual helicase functions in salinity stress tolerance by maintaining photosynthesis and antioxidant machinery in rice (*Oryza sativa L*. cv. IR64). *Plant J.* 76 115–127. 10.1111/tpj.12277 23808500

[B52] Weitbrecht K. Müller K. Leubnermetzger G. (2011). First off the mark: early seed germination. *J. Exp. Bot.* 62 3289–3309. 10.1093/jxb/err030 21430292

[B53] Xie Z. Zhang Z. L. Hanzlik S. Cook E. Shen Q. J. (2007). Salicylic acid inhibits gibberellin-induced alpha-amylase expression and seed germination via a pathway involving an abscisic-acid-inducible *WRKY* gene. *Plant Mol. Biol.* 64 293–303. 10.1007/s11103-007-9152-0 17390108

[B54] Yamaguchi S. (2008). Gibberellin metabolism and its regulation. *Annu. Rev. Plant Biol.* 59 225–251. 10.1146/annurev.arplant.59.032607.092804 18173378

[B55] Yano K. Aya K. Hirano K. Ordonio R. L. Ueguchitanaka M. Matsuoka M. (2015). Comprehensive gene expression analysis of rice aleurone cells: probing the existence of an alternative gibberellin receptor. *Plant Physiol.* 167 531–544. 10.1104/pp.114.247940 25511432 PMC4326742

[B56] Yin C. Wang X. Wu Q. Deng L. Li R. (2009). Effects of GA on seed germination and seedling growth of rice under salt stress. *J. Anhui Agric. Sci.* 37 6389–6390. 10.3969/j.issn.0517-6611.2009.14.032

[B57] Yin C. Wu Q. Zeng H. Xia K. Xu J. Li R. (2011). Endogenous auxin is required but supraoptimal for rapid growth of rice (*Oryza sativa* L.) seminal roots, and auxin inhibition of rice seminal root growth is not caused by ethylene. *J. Plant Growth Regul.* 30 20–29. 10.1007/s00344-010-9162-z

