# Supplementary material for: Salinity Inhibits Rice Seed Germination by Reducing α-Amylase Activity via Decreased Bioactive Gibberellin Content
Source: Front Plant Sci. 2018 Mar 5;9:275. doi: 10.3389/fpls.2018.00275 (PMC5845124; doi:10.3389/fpls.2018.00275)
Supplement: Supplementary file 1 [file Table_1.DOC]

**Supplementary Material**

**Supplementary Table 1.** Primers used for quantitative real-time PCR in this study.

| Gene | Forward primer 5' → 3' | Reverse primer 5' → 3' |
| --- | --- | --- |
| *OsCPS1* | TCAAGAGACACCGCCAGTTC | ACAGTGCATGACCCTGGATG |
| *OsKS1* | CTCGATCAGCTGCCATTTGC | GCGGGCTTCAGACAATTCAC |
| *OsKO1* | AACCCGCCTCCAGCTGTT | CAACTACGGAAGAAGCCCCG |
| *OsKAO* | CTCCTTCGTGTCCTTCCGTC | ACCACAGCTGAACCTTCCAC |
| *OsGA20ox1* | TTCTTCCTCTGCCCGGAGAT | CATGTCGGCCCTGTAGTGG |
| *OsGA3ox2* | CTTCTGTGACGTGATGGAGGAG | CTCAAGAACAACCTCAGCAACTC |
| *OsGA2ox1* | ACCCGCAGATCCTTAGCTTG | TGAACCCACATCTCCTTGCC |
| *OsGA2ox2* | TGTTTGGTTGAGGGGTGAGT | CATTTTCCCCGATCAGTTGGT |
| *OsGA2ox3* | ACTCGTTGCAGGTTCTGACC | GTGGCAATGGTGCAATCCTC |
| *OsGA2ox5* | GATGCCCATTACCGGAGCTT | AAGCGGTGCAGTCCAATCTT |
| *OsGA2ox6* | GGCTATCGGTGGCCTACTTC | TTGTCCTGACGTCTTCCTGC |
| *OsGA2ox9* | AGCACAGGGTGATGACGAAC | CCTTGTAAGGGGAAGGCTCC |
| *OsAmy1A* | TTTCGGTCCTCATCGTCCTCC | TCCACGACTCCCAGTTGAATC |
| *OsAmy1C* | TGGTATCGATCAGAAACCGGC | GTCCGACCTTCGTGATGACC |
| *OsAmy3C* | AAGCATTCCACCACAATGAGC | AGGAAGTTGTACCACCCACC |
| *OsAmy3E* | TCACCCTGTGTTGTGTCGTT | AAAGTTGTACCACCCGCCTT |
| *OsACTIN* | CTGACGGAGCGTGGTTACTCAT | TCATAGTCCAGGGCGATGTAGG |
